# Shared and Distinct Epigenetic Profiles in Primary Age‐Related Tauopathy and Alzheimer’s Disease: Insights into Tangle Formation and Spread

**DOI:** 10.1002/alz.092601

**Published:** 2025-01-03

**Authors:** Anil R Wadhwani, David Goldberg, David A Wolk, Eddie B Lee, Kurt W. Farrell, John F. Crary, David A. Bennett, Philip L. De Jager, Julie A. Schneider, Wanding Zhou, Corey T McMillan, Part Working Group

**Affiliations:** ^1^ University of Pennsylvania, Philadelphia, PA USA; ^2^ Icahn School of Medicine at Mount Sinai, New York, NY USA; ^3^ Rush University Medical Center, Chicago, IL USA; ^4^ Columbia University Irving Medical Center, New York, NY USA; ^5^ Children’s Hospital of Philadelphia, Philadelphia, PA USA; ^6^ Penn Frontotemporal Degeneration Center, Department of Neurology, Perelman School of Medicine, University of Pennsylvania, Philadelphia, PA USA

## Abstract

**Background:**

Alzheimer’s disease (AD) is characterized by neocortical dissemination of neurofibrillary tangles (NFTs) while primary age‐related tauopathy (PART) has NFTs largely confined to the hippocampus and adjacent structures. Thus, PART and AD represent two extremes of a spectrum of NFT spread. We investigated epigenetic mechanisms of interindividual variation in NFT spread.

**Method:**

We evaluated DNA methylation (DNAm) in frontal cortex by Infinium EPIC BeadChip array in the multi‐center PART Working Group cohort (PWG, N = 398), controlling for age, sex, and batch effects. Using SeSAMe and a false discovery rate of p<0.05, we identified differentially methylated positions (DMPs) associated with PART pathology quantitated from immunohistochemically‐stained sections. To assess amyloid effects, we compared this set with DMPs associated with neuritic amyloid plaque using the CERAD score. We identified novel DMPs not previously implicated in AD. We validated our findings in the Religious Orders Study and Memory and Aging Project cohort (ROSMAP, N = 707). We then related DMPs to differential expression of linked genes and performed enrichment analyses using KnowYourCG.

**Result:**

We identified DMPs associated with NFTs [PWG: 8 novel / 8 total; ROSMAP: 31 novel / 68 total]. In the PWG cohort, of the 550 total [539 novel] DMPs that associated with CERAD score, there was no overlap with NFT‐associated DMPs. In the ROSMAP cohort, 57 total [3 novel] DMPs are associated with both NFTs and diagnosis of PART vs. AD and are linked to genes related to T‐cells and axonal transport. In contrast, the 11 total [10 novel] DMPs associated with NFTs alone are linked to genes related to synaptic signaling, heparin sulfate proteoglycan biosynthesis, and microtubule architecture.

**Conclusion:**

DNA methylation distinguishes PART and AD brain. We identify tau‐specific DMPs that account for variance in NFT burden among aging individuals, both in the absence and presence of amyloid plaques in PART and AD, respectively. In PART, tau‐DMPs are fully orthogonal to the set of amyloid‐DMPs. In contrast, in AD, the tau‐DMPs have both amyloid‐associated and amyloid‐independent subsets with separate gene enrichment profiles. Therefore, distinct epigenetic events may drive pathology within the limbic system compared to those that drive tau spread to neocortex.

